# Effects of multidimensional life management on healthy behavior in polycystic ovary syndrome patients: A randomized controlled trial

**DOI:** 10.3389/fpsyg.2022.918991

**Published:** 2022-08-18

**Authors:** Yunmei Guo, Ying Liu, Xin Yan, Rui Ding, LianHong Wang

**Affiliations:** ^1^Nursing Department, Affiliated Hospital of Zunyi Medical University, Zunyi, China; ^2^Nursing College, Zunyi Medical University, Zunyi, China

**Keywords:** transtheoretical model, health behaviors, polycystic ovary syndrome, diet, exercise

## Abstract

**Objective:**

To confirm the effects of a transtheoretical model (TTM) based on multidimensional life management on healthy behavior in patients with polycystic ovary syndrome (PCOS).

**Methods:**

In total, eighty eligible patients were recruited from March 2021 to June 2021 and randomly assigned to either the intervention (*n* = 40) or control (*n* = 40) groups. Outcome measures include health-promoting behavior, self-efficacy, anthropometrics, and the number of unplanned outpatient admissions. Data were collected at baseline, 3, and 6 months after the intervention. The chi-square test, rank-sum test, *t*-test, and repeated measurement analysis of variance (ANOVA) were used to analyze the data.

**Results:**

In total, sixty-six participants completed the study: 35 participants in the intervention group and 31 participants in the control group. After 6 months of intervention, the healthy behavior level of patients with PCOS increased from moderate [health-promoting lifestyle profile (HPLP)-II score of 105.37 ± 12.57] to good (156.94 ± 19.36) in the intervention group; and there was no change observed in the control group. In addition, the total self-efficacy score (*p* < 0.001), PCOS-related unplanned outpatient admissions (*p* = 0.008), waist circumference (WC) (*p* = 0.016), and body mass index (BMI) (*p* = 0.011) were found to have a significant difference in the intervention group at 6 months. Meanwhile, repeated measures analysis of variance showed a significantly greater improvement in health-promoting behavior and self-efficacy over time in the intervention group than in the control group (*p* < 0.001).

**Conclusion:**

The transtheoretical model based on multidimensional life management positively affected healthy behavior, self-efficacy, the number of unplanned outpatient admissions, and anthropometrics in patients with PCOS.

**Clinical Trial Registration:**

www.chictr.org.cn, ChiCTR2000034572.

## Introduction

Polycystic ovary syndrome (PCOS) is a common endocrine disorder, affecting 4–22.5% of women of reproductive age (Skiba et al., 2018). The main features of PCOS include hyperandrogenism, menstrual dysfunction, and anovulatory and polycystic ovaries (Fauser et al., 2012; Almenning et al., 2015). Studies in patients with PCOS have shown that 50–80% are overweight or obese (Dumesic et al., 2015). Furthermore, PCOS increases the risk of additional complications, such as type 2 diabetes, metabolic syndrome, and cardiovascular diseases. To date, the specific etiology of PCOS remains unknown. A recent study (Carrie et al., 2021) reported that PCOS increases the financial burden of patients; if combined with reproductive endocrine diseases (menstrual dysfunction/abnormal uterine bleeding, hirsutism, and infertility), the total economic burden of PCOS was approximately US $8 billion per year in 2020. Treatment for PCOS includes lifestyle interventions [dietary and/or physical activity (PA)], as well as surgical and pharmacological options. Lifestyle management is an initial treatment strategy with a low treatment cost, which reduces the financial burden of patients. Furthermore, the international PCOS guidelines recommend lifestyle management as the first-line treatment for PCOS (Teede et al., 2018; Lim et al., 2019).

Although lifestyle management is the simplest and the most economical way to avoid invasive operations and long-term pharmacological therapy, poor health behavior (poor dietary intakes and lower total PA) is a common challenge in lifestyle intervention research (Liu et al., 2021). Compared with women without PCOS, those with PCOS were reported to be less active, with an increase in their sedentary time by 30 min/day, and were less likely to engage in PA (PCOS: 48 vs. non-PCOS: 64%) (Moran et al., 2013; Banting et al., 2014). In addition, many studies (Ahmadi et al., 2013; Huijgen et al., 2015; Hosseini et al., 2017; Noormohammadi et al., 2022) have shown that patients with PCOS have a reduced quality of diet, increased energy intake, and decreased PA levels compared with patients without PCOS. However, the mechanism of poor diet and exercise behavior in patients with PCOS remains unclear; the complex interaction between genetic susceptibility and environmental determinants may play a role in this (Kazemi et al., 2022). In addition, the COVID-19 epidemic blockade period also negatively impacted PA and diet behavior (Ammar et al., 2020; Trabelsi et al., 2021). Thus, breaking spatial constraints of intervention sites in the context of the COVID-19 epidemic is critical in improving healthy behaviors in patients with PCOS.

A previous study indicated that incorporating a theory model is more beneficial than non-theory-driven methodologies to nudge healthy behavior (Michie et al., 2013). However, few studies have used theoretical models to implement healthy behavior management in patients with PCOS. The transtheoretical model (TTM) (Mozhdeh et al., 2019) is the most common health behavior model, widely proposed to change behavior. The TTM views behavioral change in health as a gradual, continuous, and dynamic process in a series of stages based on the individual's willingness to adopt the change (Prochaska and Velicer, 1997). The stage of change (SOC) describes an individual's current intentions and participation in health-related target behaviors (Norcross et al., 2011; Mastellos et al., 2014). The model is comprised of five stages: pre-contemplation, contemplation, preparation, action, and maintenance. Some studies have found that intervention measures based on the TTM model can better meet the needs of patients' behavioral changes to effectively promote the improvement of healthy behavior (Joo et al., 2015; Selcuk-Tosun and Zincir, 2019; VilamalaOrra et al., 2021). However, there are few studies to provide tailored intervention according to the stage of behavior change for patients with PCOS. The change and maintenance of patients' healthy behaviors is a complex process and not a single continuous behavior change trajectory; therefore, it is necessary to provide patients with PCOS with interventions that match the stage of behavior change based on TTM and to dynamically assess the stage of behavior change in patients to improve their poor health behaviors.

We conducted a randomized clinical trial of patients with PCOS to address the poor healthy behavior in the life management programs for patients with PCOS. This study tested the hypothesis that multidimensional life management based on TTM can effectively improve patients' health behaviors, self-efficacy, anthropometric indicators, and the number of unplanned outpatient admissions.

## Methods

### Participants and setting

Patients who visited the outpatient department of the Affiliated Hospital of Zunyi Medical University from March 2021 to June 2021 were consecutively included in this study. Women of reproductive age (18–45 years) who fulfilled at least two of the three Rotterdam Criteria for the diagnosis of PCOS (such as oligo-ovulation or anovulation, hyperandrogenism, and polycystic ovaries that were confirmed *via* ultrasound) (The Rotterdam ESHRE/ASRM-sponsored PCOS Consensus Workshop Group, 2004) and knew how to use WeChat were included. Patients who refused to participate, those who were unable to read and/or understand the questionnaires provided, and patients who refused to sign an informed consent form were excluded from the study.

### Sample size and randomization

The Power Analysis and Sample Size (PASS) software (NCSS Statistical Software) was used to calculate the sample size. According to Zhihua et al. (2017), when fasting insulin was used as the calculation index, the sample size of the two groups of patients was set to be the same with alpha = 0.01 and power = 80%. The total sample size of the two groups combined was calculated to be 66 cases (*n* = 33 in each group). Considering the sample follow-up loss caused by uncontrollable factors, we increased the sample size by 20%. Finally, a total sample size of 80 was determined: 40 patients in the intervention group and 40 in the control group.

An independent researcher prepared the randomization sequence using a computer-generated block randomization list for 1:1 allocation of participants into the two groups (intervention and control groups) with a block size of four. Following the baseline data collection process, participants were randomly assigned to the groups by a researcher who was blinded to the study design.

### Procedures

Patients with PCOS potentially eligible for the study were identified by gynecologists in outpatient clinics based on diagnostic criteria. If the patient met the inclusion criteria, the investigator informed them of the purpose, procedures, and benefits of the study. Data collection began after informed consent was obtained from the patients. Participants had attended the outpatient clinic for standardized screening, conducted by two researchers who were blinded to group allocation. Participant measurements were collected at baseline, 3, and 6 months. The differences in results between the intervention group and the control group were evaluated at different time points to examine the long-term effectiveness of the TTM-based intervention program.

### Interventions

#### Intervention group

The intervention was conducted by a research team consisting of two dietitians, three gynecologists, two advanced nurses, a nutritionist, and physical therapists. The medical staff member guided the patients to join the WeChat group during the first visit. The TTM-based intervention included the following sessions:

1) Preaction stage: this stage includes three parts: the first part—titled “*Knowledge and information about PCOS*”—consisted of two lectures, namely, “Basic knowledge of PCOS” and “The benefits of a healthy lifestyle for patients with PCOS” *via* the Tencent conference. The second part—titled “*WeChat-based motivational interviews*”—consisted of three lectures, namely, “Establishing rapport and understanding the current lifestyle practice with patients in PCOS,” “Eliciting and establishing autonomous motivation for a change in exercise and diet behavior,” and “Collaboratively identifying behavior changes strategies which the individual recognized.” The third part—titled “*Individualized behavior change strategy*”—consisted of diet and exercise programs that met the physical needs of the patients.
2) Action stage: this stage includes three parts. The first part—titled “*Diet and exercise diary*”—required participants to record their exercise and diet (such as the diet type, amount, and whether to add meals; the exercise type, frequency, time, and heart rate). The second part—titled “*Peer support*”—was a WeChat group. Patients with PCOS who maintain healthy lifestyles for the long term shared their successful experiences in maintaining a healthy lifestyle and helped patients enhance their confidence in behavior change. The third part—titled “*Brief motivational interviews*”—occurred at least every 2–3 days but not more than two times daily to enhance the awareness of behavior change during the period according to the participant feedback on dietary or exercise records.
3) Maintain stage: this stage includes two parts. The first part—titled “*Diet and exercise diary*”—and the second part—titled “*Brief motivational interviews*”—were as detailed in the Action stage.

#### Follow-up booster intervention

The trained researchers followed-up the intervention group individually *via* WeChat or phone throughout the study period. Researchers delivered brief follow-up intervention messages more intensively as information was demanded by the participant (usually not <1 time per 2–3 days and not more than two times per day) during the pre-action and action stages. The frequency of delivering messages through WeChat or mobile phone was interactive, which depended on the participants' stage of behavior change. Patients took several sessions of chats per week *via* WeChat. When the healthy behavior of diet or exercise entered the maintenance stage, minimal messages were sent, which merely followed the participants' progress and responded to their questions to maintain contact.

#### Control group

Participants in the control group only received routine care, which consisted of unstructured and straightforward patient education about health behavior.

### Measures

#### Sociodemographic characteristics

The sociodemographic characteristics questionnaire obtained information on age, education level, living residence, marital status, occupation, weight, height, body mass index (BMI), and waist circumference (WC). Weight was measured with light clothing and without shoes. Height was measured without shoes using a stadiometer. The BMI was calculated based on height and weight. WC was measured in centimeters using plastic tape at the midpoint between the costal margin and the iliac crest in the mid-axillary line in the standing position at the end of a gentle expiration.

#### Healthy behavior

We evaluated health behavior using a health-promoting lifestyle profile (HPLP)-II. This scale consists of six dimensions and 52 items, such as health responsibility (nine items), nutrition (nine items), PA (eight items), interpersonal relationships (nine items), stress management (eight items), and spiritual growth (nine items). Each item in the questionnaire was answered using a four-point Likert scale with 1, 2, 3, and 4 corresponding to never, sometimes, often, and routinely, respectively. Total scores ranged from 52 to 208, with higher scores representing better health-promoting behavior (Tanjani et al., 2016). The HPLP-II scale has good internal consistency; the Cronbach's alpha of the scale is 0.94, and the six subscales are 0.79–0.87 (Walker et al., 1995). The overall HPLP-II score is divided into four levels: 52–90 as poor, 91–139 as medium, 140–168 as good, and 169–208 as excellent (Walker et al., 1987).

#### Self-efficacy

We assessed self-efficacy using the Self-Efficacy Scale for Chronic Disease (SECD6), which consists of six items on a 10-step Likert Scale, ranging from 1 (not fully confident) to 10 (completely confident). This scale was interpreted by calculating the average score for at least four of the six items, thus allowing for up to two items with missing answers. Means ranged from 1 to 10, indicating higher self-efficacy (Chenli et al., 2017).

#### Number of unplanned outpatient admissions

The number of unplanned outpatient admissions was measured using a questionnaire that asked participants whether they had missed or mistakenly taken medicine to cause irregular menstruation or bleeding.

### Ethical considerations

The study received official approval from the Institutional Review Board (IRB) of the Affiliated Hospital of Zunyi Medical University (NO. KLLY-2020-134). Informed consent was obtained from all the participants, and procedures were conducted according to the Declaration of Helsinki. All eligible participants had the right to withdraw without any adverse effects on clinical care.

### Statistical analysis

We used SPSS Statistics Version 18.0 to analyze the data. The significance level was set at 0.05 (two-tailed). The mean ± standard deviation (SD) baseline data conformed to the normal distribution, and the percentage change data conformed to the non-normal distribution. A *T*-test was used to compare the average distribution data of continuous variables between the two groups of patients. The Mann–Whitney *U*-test and the chi-square test were used to compare the intervention and control groups for continuous variables without normal distribution or discrete quantitative variables. Researchers performed repeated-measures analysis of variance for comparisons among the different time points.

## Results

**Figure 1:**
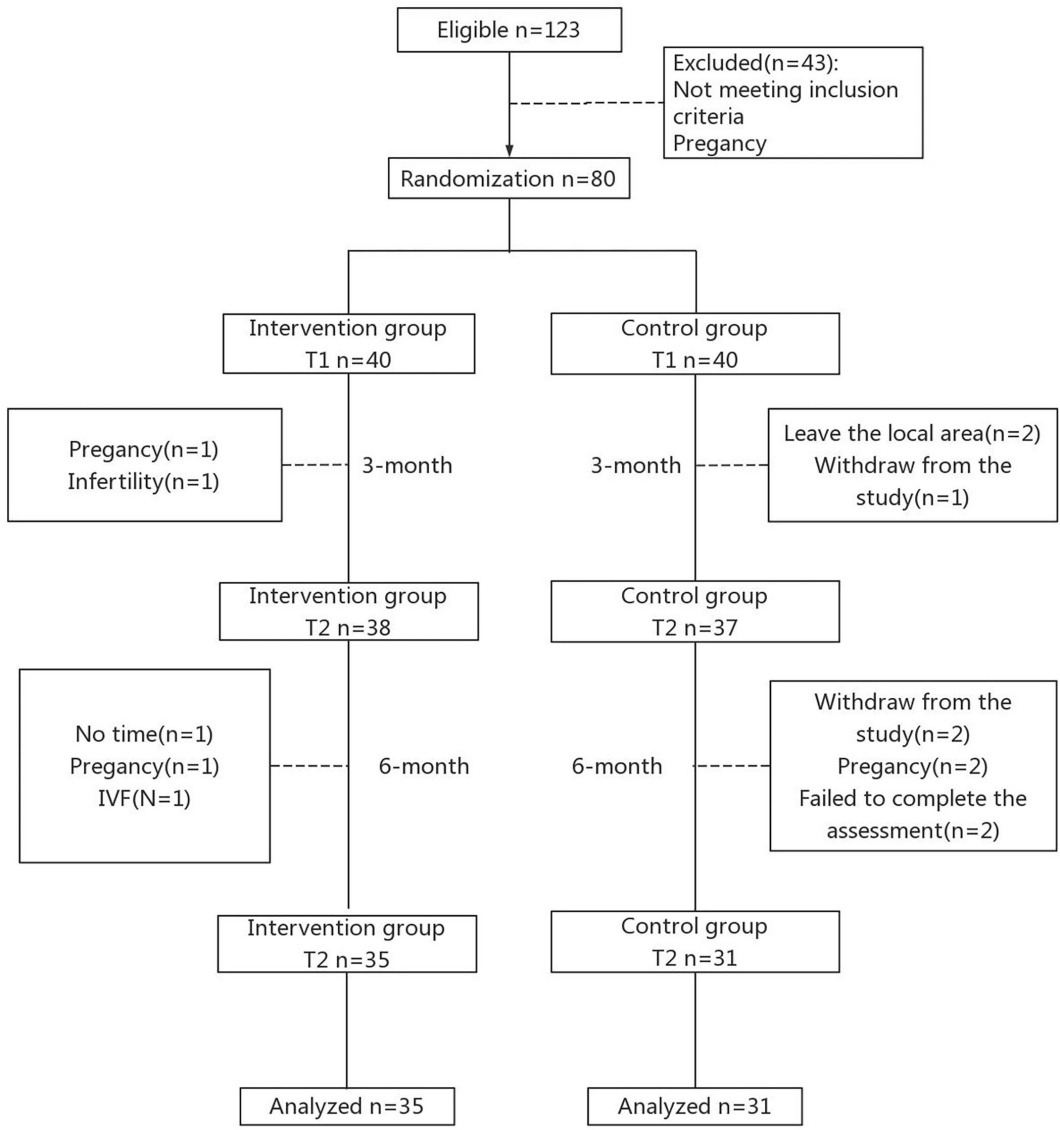
Consort flow diagram.

Of the 123 participants enrolled in the trial between March 2021 and June 2021, 66 completed the study, including 35 participants in the intervention group and 31 in the control group. Figure 1 shows the participant flow throughout the study. There were no statistically significant differences in the sociodemographic characteristics between participants in the intervention group and the control group (*p* > 0.05; Table 1).

**Table 1:** Comparison of general data between two groups of patients.

**Variable**	**Categories**	**Intervention group (*N* = 35)**	**Control group (*N* = 31)**	**χ^2^/t/Z**	***P*-Value**
Age (x ± s)		24.95 ± 4.02	25.98 ± 4.05	−0.35*	0.73
BMI (x ± s)		25.86 ± 2.64	25.45 ± 2.42	0.66	0.51
WC (x ± s)		87.44 ± 5.92	86.85 ± 4.63	0.44	0.66
Ethnic group [*N* (%)]	Han-Nationality	25 (71.43)	22 (70.97)	0.002^▴^	0.967
	Ethnic minority	10 (28.57)	9 (29.03)		
Living residence [*N* (%)]	City	13 (37.14)	11 (35.48)	0.020^▴^	0.889
	Countryside	22 (62.86)	20 (64.52)		
Marital status [*N* (%)]	Single	19 (54.29)	18 (58.06)	0.256^▴^	0.613
	Married	15 (42.86)	13 (41.94)		
	Widowed/divorced	1 (2.86)	0 (0.00)		
Education [*N* (%)]	Middle school	12 (34.29)	10 (32.26)	0.067^▴^	0.796
	High school	4 (11.43)	7 (22.58)		
	Junior college	5 (14.29)	2 (6.45)		
	College	14 (40.00)	12 (38.71)		
Occupation [*N* (%)]	Employed	13 (37.14)	11 (35.48)	0.735^▴^	0.391
	Unemployed	7 (20.00)	3 (9.68)		
	Student	8 (22.86)	7 (22.58)		
	Other	7 (20.00)	10 (32.26)		
Years of PCOS [*N* (%)]	<1years	14 (40.00)	16 (51.61)	−1.198^Δ^	0.231
	1-3years	17 (48.57)	14 (45.16)		
	4-6years	2 (5.71)	1 (3.23)		
	> 7years	2 (5.71)	0 (0.00)		
Whether there is a need for pregnancy	Yes	17 (48.57)	20 (64.52)	1.697^▴^	0.193
	No	18 (51.43)	11 (35.48)		
Menstrual disorder	Yes	21 (60.00)	18 (58.06)	0.025^▴^	0.873
	No	14 (40.00)	13 (41.94)		

*, t; ▴, χ^2^; Δ, z.

**Figure 2:**
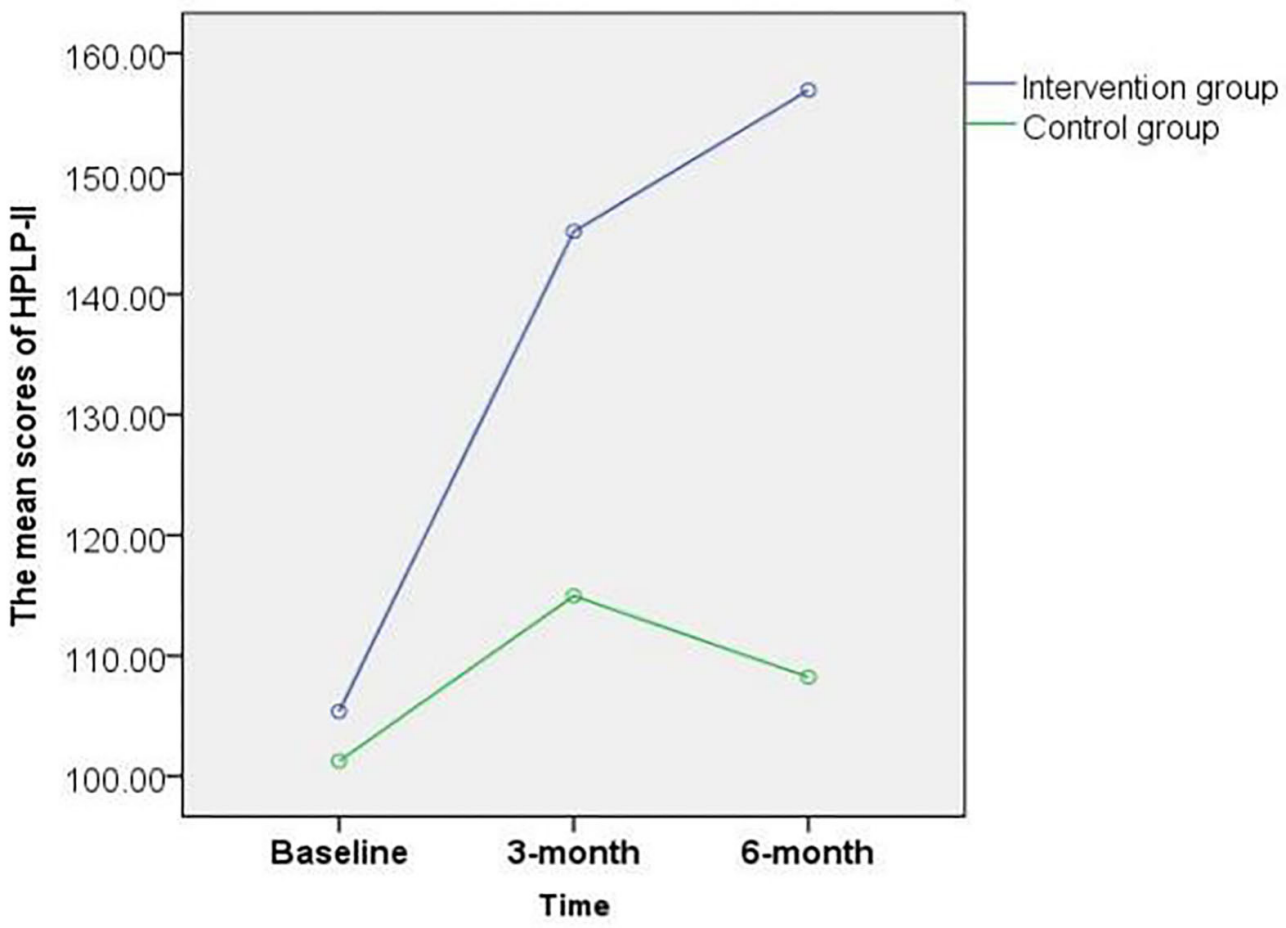
Total score changes in healthy behavior at baseline, third, and sixth month post-intervention among the PCOS patients.

There was no significant difference in the HPLP-II score between the intervention and control groups at baseline (*p* > 0.05). After 6 months of intervention, the healthy behavior level of patients with PCOS increased from moderate (105.37 ± 12.57) to good (156.94 ± 19.36) in the intervention group. The interaction between time and groups was statistically different (*p* < 0.001, Figure 2). However, there was no significant change in the control group (101.26 ± 15.21 to 108.23 ± 15.32) at the different time points. The scores of health responsibility (22.52 ± 5.03 vs. 18.26 ± 6.01), nutrition (27.69 ± 4.09 vs. 20.61 ± 5.12), PA (25.34 ± 4.26 vs. 16.09 ± 4.89), interpersonal relationships (28.11 ± 4.34 vs. 19.58 ± 6.89), spiritual growth (28.23 ± 3.42 vs. 20.94 ± 6.36), and stress management (23.91 ± 3.92 vs. 16.97 ± 5.89) in the intervention group were significantly higher than those in the control group (*p* < 0.001, Table 2).

**Table 2:** Comparison of HPLP-II score at baseline, third, and sixth month.

**Measured parameter**	**Baseline**	**3-Months**	**6-Months**	***F*_(time)_**	***F*_(group)_**	*F*_(time*group)_
**Total HPLP scorers**				73.251***	66.904***	23.951***
Intervention group (*n* = 35)	105.37 ± 12.57	145.20 ± 17.19	156.94 ± 19.36			
Control group (*n* = 31)	101.26 ± 15.21	114.97 ± 17.64	108.23 ± 15.32			
*t*-value	1.203	7.044	7.055			
*p*-value	0.234	0.00***	0.00***			
**Healthy responsibility**				30.371***	18.010***	4.742*
Intervention group (*n* = 35)	15.14 ± 3.31	22.51 ± 5.15	22.52 ± 5.03			
Control group (*n* = 31)	15.09 ± 3.17	18.29 ± 3.74	18.26 ± 6.01			
*t*-value	0.058	3.769	3.069			
*p*-value	0.954	0.00***	0.003			
**Nutrition**				55.457***	31.606***	18.303***
Intervention group (*n* = 35)	17.37 ± 4.24	25.51 ± 4.26	27.69 ± 4.09			
Control group (*n* = 31)	17.81 ± 3.53	19.97 ± 4.03	20.61 ± 5.12			
*t*-value	−0.446	5.415	6.242			
*p*-value	0.657	0.00***	0.00***			
**Physical activity**				97.203***	77.739***	27.507***
Intervention group (*n* = 35)	13.00 ± 2.49	21.80 ± 5.38	25.34 ± 4.26			
Control group (*n* = 31)	12.29 ± 2.95	14.93 ± 3.74	16.09 ± 4.89			
*t*-value	1.059	5.943	8.216			
*p*-value	0.294	0.00***	0.00***			
**Interpersonal relations**				12.549***	30.804***	11.847***
Intervention group (*n* = 35)	20.74 ± 4.02	25.71 ± 3.82	28.11 ± 4.34			
Control group (*n* = 31)	19.94 ± 5.09	21.81 ± 5.15	19.58 ± 6.89			
*t*-value	0.719	3.526	6.091			
*p*-value	0.475	0.001	0.00***			
**Spiritual growth**				19.686***	56.376***	9.316**
Intervention group (*n* = 35)	20.54 ± 4.29	27.26 ± 3.96	28.23 ± 3.42			
Control group (*n* = 31)	19.71 ± 4.05	21.26 ± 4.54	20.94 ± 6.36			
*t*-value	0.808	5.737	5.896			
*p*-value	0.422	0.00***	0.00***			
**Stress management**				9.669***	44.243***	9.575***
Intervention group (*n* = 35)	18.57 ± 3.48	23.34 ± 4.15	23.91 ± 3.92			
Control group (*n* = 31)	17.67 ± 3.41	18.71 ± 3.54	16.97 ± 5.89			
*t*-value	1.052	4.845	5.702			
*p*-value	0.297	0.00***	0.00***			

*p < 0.05; **p < 0.01; ***p < 0.001.

**Figure 3:**
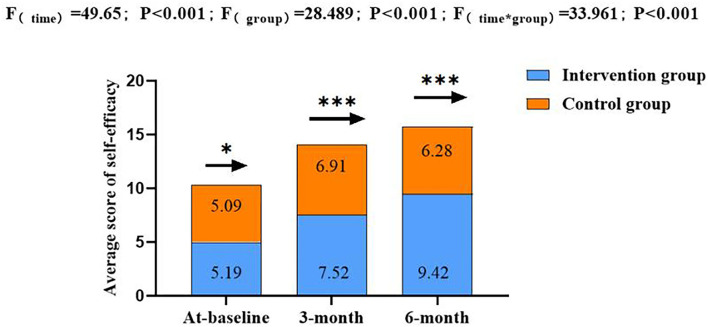
Total score changes in self-efficacy at baseline, third, and sixth month post-intervention among the PCOS patients. *p > 0.05, **p < 0.01, ***p < 0.001; *Repeated measurement analysis of variance.

The groups had no significant difference in self-efficacy at baseline (*p* > 0.05). The interaction between time and groups was statistically different (*p* < 0.05). Regarding the self-efficacy score, the intervention group showed a significant increase from 5.01 ± 1.86 to 9.50 ± 0.58 (*p* < 0.01); however, no significant difference was observed in the control group (*p* > 0.05, Figure 3).

There was no significant difference in BMI and WC at baseline (*p* > 0.05). However, the interaction between the time and groups was statistically different (*p* < 0.05). The BMI in the intervention group showed a significant decrease from baseline (25.86 ± 2.64 kg/m^2^) to that at 6 months (22.27 ± 3.01 kg/m^2^), and WC decreased significantly from baseline (87.44 ± 5.92 cm) to that at 6 months (81.07 ± 7.08 cm); however, no significant differences at different time points were observed in the control group (Table 3).

**Table 3:** Comparison of BMI and WC at baseline, third, and sixth month.

**Items**	**At-Baseline**	**3-Month**	**6-Month**	***F*_time_**	***F*_group_**	*F*_(time*group)_
**BMI**				14.178***	5.464*	3.77*
Intervention group (*n* = 38)	25.86 ± 2.64	23.01 ± 1.64	22.27 ± 3.01			
Control group (*n* = 36)	25.45 ± 2.42	24.46 ± 2.54	24.33 ± 3.40			
*t*-value	0.662	−2.16	−2.61			
*p*-value	0.511	0.034	0.011			
**WC**				7.64**	4.902*	2.452 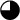
Intervention group (*n* = 38)	87.44 ± 5.92	84.54 ± 5.85	81.07 ± 7.08			
Control group (*n* = 36)	86.85 ± 4.63	86.92 ± 6.59	85.09 ± 6.06			
*t*-value	0.445	−1.552	−2.465			
*p*-value	0.658	0.126	0.016			

*
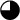
P > 0.05; *p < 0.05; **p < 0.01; ***p < 0.001*.

Table 4 shows that only three patients in the intervention group had unplanned outpatient admissions during the intervention period, while 11 patients did in the control group. The unplanned outpatient admission rate was 8.57 and 35.48% in the intervention and control groups, respectively. The unplanned outpatient admission rates showed significant differences at 3 months (*p* = 0.045) and 6 months (*p* = 0.008) between the intervention and control groups.

**Table 4:** The change of unplanned admission at third and sixth month among PCOS patients.

	**3-Month**		**6-Month**		**Unplanned admission rate**
	**Yes**	**No**	**Yes**	**No**	***N* (%)**
	***N* (%)**	***N* (%)**	***N* (%)**	***N* (%)**	
Intervention group (*N* = 35)	0 (0.00)	35 (100)	3 (8.57)	32 (91.43)	8.57
Control group (*N* = 31)	5(16.13)	26 (83.87)	11 (35.48)	20 (64.52)	35.48
χ^2^	4.022		7.124		
*P*-value	0.045		0.008		

## Discussion

In total, eighty subjects were included in the study sample at the beginning of the intervention phase; however, only 66 (82.50%) completed the study, with 35 patients in the intervention group and 31 in the control group. Our study indicated that a 6 month TTM-based stage-matched intervention could significantly improve healthy behavior, self-efficiency, and unplanned outpatient admissions. In addition, the intervention program promoted a decrease in BMI and WC.

During the 6 month intervention period, the increase in scores of healthy behaviors in the experimental group was more significant than in the control group. Patients' health behaviors in the intervention group transferred from a moderate to good level, which is consistent with previous studies (Xu et al., 2020). The TTM-based tailored intervention program is suggested to be the main reason for the successful promotion of health behaviors in this study. Most previous studies (Jiskoot et al., 2020; Moran et al., 2020) systematically provided lifestyle interventions for patients with PCOS; they did not provide personalized measures according to the stage of the patients' behavior and ignored the dynamic process of behavioral changes. This study provides matching intervention measures for patients according to their behavior change stage (for example, when the patient is in the pre-contemplation or contemplation, the main purpose of this stage is to make the patient understand the relevant knowledge and importance of life management, promote the awakening of patients' behavioral awareness, and ensure that they are fully prepared for entering the action stage). This was expected to enhance the motivation and enthusiasm of patients for behavior change, thus increasing their participation rate. As a result, we promoted an improvement in patients' health behaviors. A previous study (Nicolson et al., 2018) reported that most patients could not successfully maintain their behavioral changes when they tried to take action for the first time, and the same phenomenon inevitably occurred in the intervention group in this study. However, in this study, researchers understood the causes of retrogression in patients' behavior in a timely manner and provided targeted solutions to encourage participants to return to the action period, thereby promoting the improvement of patients' health behavior.

In addition, the booster intervention (WeChat or phone follow-up) implemented during the whole intervention period played an important role in improving patients' health behavior. During the COVID-19 epidemic lockdown (Trabelsi et al., 2021), traditional face-to-face interventions were impossible to continue, which prevented us from being able to respond to the diversified needs of the PCOS population. The flexibility (Donovan et al., 2015) of the booster intervention is more conducive to breaking through the space limitation of COVID-19's intervention during the epidemic. Furthermore, the booster intervention aimed to consolidate or improve the initial effect of the intervention (Tolan et al., 2009), which is a key in promoting the maintenance of the health behavior of patients with PCOS.

Improving self-efficacy is generally regarded as an essential intermediary factor in the effect of exercise interventions (Olander et al., 2013). Self-efficacy and autonomous motivation are the core psychological factors that affect the initiation and maintenance of healthy behaviors (Pelletier et al., 2017; Castillo-Mayen et al., 2020). In the present study, the TTM-based program was found to be an effective method for developing self-efficacy, resulting in regular exercise, a healthy diet, and positively enhanced health literacy. Similar to the results reported in other studies, the TTM intervention program increased the score of patients' self-efficacy (Selcuk-Tosun et al., 2019). In the present study, as a distinct aspect of life management performed in previous studies, researchers dynamically assessed the participant's diet and exercise behavior. This method might provide additional benefits for participants in maintaining their behaviors and helping them to enhance their behavioral awareness.

It is noteworthy that this study focuses on the unplanned admission of patients with PCOS to hospitals and identifies the TTM-based stage-matched program as a positive approach to lower the unplanned outpatient admission rates for the first time. In the present study, the unplanned outpatient admission rate of patients with PCOS in the experimental group (8.57%) was lower than that in the control group (35.48%). The reason for this phenomenon may be that with the healthy behavior and negative emotions regarding improving metabolic disorder relief, participants experienced a reserve in the physiological and mental system (Jiskoot et al., 2020; Oberg et al., 2020), which may explain the healthier lifestyle and the lower unplanned admission rates in our study. A previous study reported that unplanned admission would also increase the psychological burden and economic pressure (Bosco et al., 2014). However, in the current study, the unplanned outpatient admission rate of patients with PCOS did not attract the attention of researchers. Therefore, to prevent the rising cost of healthcare and decrease the risk factors of long-term complications for patients with PCOS, future research should pay more attention to the unplanned outpatient admission rate and explore more targeted strategies.

Our study has several limitations. First, it was a single-center study, making it difficult to generalize the findings. Second, we applied a non-specific scale to evaluate the healthy behavior of patients with PCOS, which could cause result bias and decrease accuracy. Finally, a passive control group has limitations (e.g., it does not account for placebo or demand effects). One of the strengths of this study is that it was the first to implement a beneficial behavior intervention based on the TTM for patients with PCOS. We focus on the dynamic evolution of behavioral intentions. Researchers used brief motivation interviews and longitudinal follow-up to nudge behavior change awareness, enhancing the healthy behavior of patients with PCOS.

## Conclusion

The TTM-based multidimensional life management model positively promotes a health behavior switch to good level. Further research is suggested to explore strategies to maintain long-term (>6 months) effects of the TTM-based multidimensional life management on poor health behavior in patients with PCOS.

## Data availability statement

The original contributions presented in the study are included in the article, further inquiries can be directed to the corresponding author.

## Ethics statement

The studies involving human participants were reviewed and approved by Institutional Review Board (IRB) of the Affiliated Hospital of Zunyi Medical University (NO. KLLY-2020-134). The patients/participants provided their written informed consent to participate in this study. Written informed consent was obtained from the individual(s) for the publication of any potentially identifiable images or data included in this article.

## Author contributions

All authors listed have made a substantial, direct, and intellectual contribution to the work and approved it for publication.

## Funding

This study was funded by the Science and Technology Department of Guizhou Province, China [Grant No. Qian Ke He (2017)5733-076].

## Conflict of interest

The authors declare that the research was conducted in the absence of any commercial or financial relationships that could be construed as a potential conflict of interest.

## Publisher's note

All claims expressed in this article are solely those of the authors and do not necessarily represent those of their affiliated organizations, or those of the publisher, the editors and the reviewers. Any product that may be evaluated in this article, or claim that may be made by its manufacturer, is not guaranteed or endorsed by the publisher.

## References

[B1] Ahmadi A. Akbarzadeh M. Mohammadi F. Akbari M. Jafari B. Tolide-Ie H. R. (2013). Anthropometric characteristics and dietary pattern of women with polycystic ovary syndrome. Indian J. Endocrinol. Metab. 17, 672–676. 10.4103/2230-8210.113759 23961484 PMC3743368

[B2] Almenning I. Rieber-Mohn A. Lundgren K. M. Shetelig L. T. Garnaes K. Moholdt T. (2015). Effects of high intensity interval training and strength training on metabolic, cardiovascular and hormonal outcomes in women with polycystic ovary syndrome: a pilot study. PLoS ONE 10, e138793. 10.1371/journal.pone.0138793 26406234 PMC4583183

[B3] Ammar A. Brach M. Trabelsi K. Chtourou H. Boukhris O. Masmoudi L.. (2020). Effects of COVID-19 home confinement on eating behaviour and physical activity: results of the ECLB-COVID19 international online survey. Nutrients 12, 1583. 10.3390/nu12061583 32481594 PMC7352706

[B4] Banting L. K. Gibson-Helm M. Polman R. Teede H. J. Stepto N. K. (2014). Physical activity and mental health in women with polycystic ovary syndrome. BMC Womens Health 14, 51. 10.1186/1472-6874-14-51 24674140 PMC3986680

[B5] Bosco J. R. Karkenny A. J. Hutzler L. H. Slover J. D. Iorio R. (2014). Cost burden of 30-day readmissions following medicare total hip and knee arthroplasty. J. Arthroplasty 29, 903–905. 10.1016/j.arth.2013.11.006 24332969

[B6] Carrie R. Anika J. Daniela M. Buyalos R. P. Azziz R. (2021). Health care-related economic burden of polycystic ovary syndrome in the United States: pregnancy-related and long-term health consequences. J. Clin. Endocrinol. Metab. 107, 575–585. 10.1210/clinem/dgab613 34546364

[B7] Castillo-Mayen R. Cano-Espejo C. Luque B. Cuadrado E. Gutierrez-Domingo T. Arenas A.. (2020). Influence of self-efficacy and motivation to follow a healthy diet on life satisfaction of patients with cardiovascular disease: a longitudinal study. Nutrients 12, 1903. 10.3390/nu12071903 32605026 PMC7400119

[B8] Chenli W. Juntao L. Lixia X. Xuemei L. Liang Z. (2017). The effect of health literacy and self-management efficacy on the health-related quality of life of hypertensive patients in a western rural area of China: a cross-sectional study. Int. J. Equity Health 16, 58. 10.1186/s12939-017-0551-9 28666443 PMC5493849

[B9] Donovan D. M. Hatch-Maillette M. A. Phares M. M. McGarry E. Peavy K. M. Taborsky J. (2015). Lessons learned for follow-up phone booster counseling calls with substance abusing emergency department patients. J. Subst. Abuse Treat. 50, 67–75. 10.1016/j.jsat.2014.10.013 25534151 PMC4305001

[B10] Dumesic D. A. Oberfield S. E. Stener-Victorin E. Marshall J. C. Laven J. S. Legro R. S.. (2015). Scientific statement on the diagnostic criteria, epidemiology, pathophysiology, and molecular genetics of polycystic ovary syndrome. Endocr. Rev. 36, 487–525. 10.1210/er.2015-1018 26426951 PMC4591526

[B11] Fauser B. C. Tarlatzis B. C. Rebar R. W. Legro R. S. Balen A. H. Lobo R.. (2012). Consensus on women's health aspects of polycystic ovary syndrome (PCOS): the Amsterdam ESHRE/ASRM-Sponsored 3rd PCOS consensus workshop group. Fertil. Steril. 97, 28–38. 10.1016/j.fertnstert.2011.09.024 22153789

[B12] Hosseini M. S. Dizavi A. Rostami H. Parastouei K. Esfandiari S. (2017). Healthy eating index in women with polycystic ovary syndrome: a case-control study. Int. J. Reprod. Biomed. 15, 575–582 10.29252/ijrm.15.9.575 29662966 PMC5894473

[B13] Huijgen N. A. Laven J. S. Labee C. T. Louwers Y. V. Willemsen S. P. Steegers-Theunissen R. P. (2015). Are dieting and dietary inadequacy a second hit in the association with polycystic ovary syndrome severity? PLoS ONE 10, e142772. 10.1371/journal.pone.0142772 26569630 PMC4646482

[B14] Jiskoot G. Dietz D. L. A. Beerthuizen A. Timman R. Busschbach J. Laven J. (2020). Long-term effects of a three-component lifestyle intervention on emotional well-being in women with polycystic ovary syndrome (PCOS): a secondary analysis of a randomized controlled trial. PLoS ONE 15, e233876. 10.1371/journal.pone.0233876 32479544 PMC7263605

[B15] Joo Y. L. Hyeoun-Ae P. Yul H. M. (2015). Transtheoretical model-based nursing intervention on lifestyle change: a review focused on intervention delivery methods. Asian Nurs. Res. 9, 158–167. 10.1016/j.anr.2015.05.001 26160246

[B16] Kazemi M. Kim J. Y. Wan C. Xiong J. D. Michalak J. Xavier I. B.. (2022). Comparison of dietary and physical activity behaviors in women with and without polycystic ovary syndrome: a systematic review and meta-analysis of 39,471 women. Hum. Reprod. Update 26, dmac023. 10.1093/humupd/dmac023 35639552 PMC9629501

[B17] Lim S. S. Hutchison S. K. Van Ryswyk E. Norman R. J. Teede H. J. Moran L. J. (2019). Lifestyle changes in women with polycystic ovary syndrome. Cochrane Database Syst. Rev. 3, D7506. 10.1002/14651858.CD007506.pub4 30921477 PMC6438659

[B18] Liu C. Zhang L. Zheng W. Liang X. Zhang L. Tian Z.. (2021). Lifestyle intervention for overweight/obese pregnant women with polycystic ovarian syndrome: lessons and challenges. Obes. Facts 14, 405–414. 10.1159/000514931 34311460 PMC8406241

[B19] Mastellos N. Gunn L. H. Felix L. M. Car J. Majeed A. (2014). Transtheoretical model stages of change for dietary and physical exercise modification in weight loss management for overweight and obese adults. Cochrane Database Syst. Rev. 5, CD008066. 10.1002/14651858.CD008066.pub3 24500864 PMC10088065

[B20] Michie S. Richardson M. Johnston M. Abraham C. Francis J. Hardeman W.. (2013). The behavior change technique taxonomy (v1) of 93 hierarchically clustered techniques: building an international consensus for the reporting of behavior change interventions. Ann. Behav. Med. 46, 81–95. 10.1007/s12160-013-9486-6 23512568

[B21] Moran L. J. Ranasinha S. Zoungas S. McNaughton S. A. Brown W. J. Teede H. J. (2013). The contribution of diet, physical activity and sedentary behaviour to body mass index in women with and without polycystic ovary syndrome. Hum. Reprod. 28, 2276–2283. 10.1093/humrep/det256 23771201

[B22] Moran L. J. Tassone E. C. Boyle J. Brennan L. Harrison C. L. Hirschberg A. L.. (2020). Evidence summaries and recommendations from the international evidence-based guideline for the assessment and management of polycystic ovary syndrome: lifestyle management. Obes. Rev. 21, e13046. 10.1111/obr.13046 32452622

[B23] Mozhdeh H. Alireza R. Firoozeh Z. Amir A. Azra D. (2019). Transtheoretical model of health behavioral change: a systematic review. Iran. J. Nurs. Midwifery Res. 24, 83–90. 10.4103/ijnmr.IJNMR_94_17 30820217 PMC6390443

[B24] Nicolson P. Hinman R. S. Kasza J. Bennell K. L. (2018). Trajectories of adherence to home-based exercise programs among people with knee osteoarthritis. Osteoarthritis Cartilage 26, 513–521. 10.1016/j.joca.2018.01.009 29360592

[B25] Noormohammadi M. Eslamian G. Malek S. Shoaibinobarian N. Mirmohammadali S. N. (2022). The association between fertility diet score and polycystic ovary syndrome: a case-control study. Health Care Women Int. 43, 70–84. 10.1080/07399332.2021.1886298 33797335

[B26] Norcross J. C. Krebs P. M. Prochaska J. O. (2011). Stages of change. J. Clin. Psychol. 67, 143–154. 10.1002/jclp.20758 21157930

[B27] Oberg E. Lundell C. Blomberg L. Gidlof S. B. Egnell P. T. Hirschberg A. L. (2020). Psychological well-being and personality in relation to weight loss following behavioral modification intervention in obese women with polycystic ovary syndrome: a randomized controlled trial. Eur. J. Endocrinol. 183, 1–11. 10.1530/EJE-20-0066 32503005

[B28] Olander E. K. Fletcher H. Williams S. Atkinson L. Turner A. French D. P. (2013). What are the most effective techniques in changing obese individuals' physical activity self-efficacy and behaviour: a systematic review and meta-analysis. Int. J. Behav. Nutr. Phys. Act. 10, 29. 10.1186/1479-5868-10-29 23452345 PMC3639155

[B29] Pelletier L. G. Guertin C. Rocchi M. (2017). “The distinctive roles of perceptions of health risks and benefits, self-efficacy, and motivation in the awareness, initiation, and maintenance of healthy behavior,” in Self: Driving Positive Psychology and Well Being, eds F. Guay, H. W. Marsh, D. M. Mcinemy, and R, G, Graven (IAP Information Age Publishing), 135–165.

[B30] Prochaska J. O. Velicer W. F. (1997). The transtheoretical model of health behavior change. Am. J. Health Promot. 12, 38–48. 10.4278/0890-1171-12.1.38 10170434

[B31] Selcuk-Tosun A. Zincir H. (2019). The effect of a transtheoretical model-based motivational interview on self-efficacy, metabolic control, and health behaviour in adults with type 2 diabetes mellitus: a randomized controlled trial. Int. J. Nurs. Pract. 25, e12742. 10.1111/ijn.12742 31090161

[B32] Skiba M. A. Islam R. M. Bell R. J. Davis S. R. (2018). Understanding variation in prevalence estimates of polycystic ovary syndrome: A systematic review and meta-analysis. Hum. Reprod. Update. 24, 694–709. 10.1093/humupd/dmy022 30059968

[B33] Tanjani P. T. Azadbakht M. Garmaroudi G. Sahaf R. Fekrizadeh Z. (2016). Validity and reliability of health promoting lifestyle profile II in the Iranian elderly. Int. J. Prev. Med. 7, 74. 10.4103/2008-7802.182731 27280010 PMC4882969

[B34] Teede H. J. Misso M. L. Costello M. F. Anuja D. Joop L. Lisa M.. (2018). Recommendations from the international evidence-based guideline for the assessment and management of polycystic ovary syndrome. Clin. Endocrinol. 110, 364–379. 10.1016/j.fertnstert.2018.05.004 30033227 PMC6939856

[B35] The Rotterdam ESHRE/ASRM-sponsored PCOS Consensus Workshop Group (2004). Revised 2003 consensus on diagnostic criteria and long-term health risks related to polycystic ovary syndrome (PCOS). Hum. Reprod. 9, 41–47. 10.1093/humrep/deh098 14688154

[B36] Tolan P. H. Gorman-Smith D. Henry D. Schoeny M. (2009). The benefits of booster interventions: evidence from a family-focused prevention program. Prev. Sci. 10, 287–297. 10.1007/s11121-009-0139-8 19513845

[B37] Trabelsi K. Ammar A. Masmoudi L. Boukhris O. Chtourou H. Bouaziz B.. (2021). Sleep quality and physical activity as predictors of mental wellbeing variance in older adults during COVID-19 lockdown: ECLB COVID-19 international online survey. Int. J. Environ. Res. Public Health 18, 4329. 10.3390/ijerph18084329 33921852 PMC8073845

[B38] VilamalaOrra M. VaquéCrusellas C. FoguetBoreu Q. Guimerà G. M. Del R. S. R. (2021). Applying the stages of change model in a nutrition education programme for the promotion of fruit and vegetable consumption among people with severe mental disorders (DIETMENT). Nutrients 132105. 10.3390/nu13062105 34205403 PMC8235404

[B39] Walker S. N. Sechrist K. R. Pender N. J. (1987). The health-promoting lifestyle profile: development and psychometric characteristics. Nurs. Res. 36, 76–81 10.1097/00006199-198703000-00002 3644262

[B40] Walker S. N. Sechrist K. R. Pender N. J. (1995). Health Promotion Model - Instruments to Measure Health Promoting Lifestyle. Available online at: http://hdl.handle.net/2027.42/85349

[B41] Xu Z. Geng J. Zhang S. Zhang K. Yang L. Li J.. (2020). A mobile-based intervention for dietary behavior and physical activity change in individuals at high risk for type 2 diabetes mellitus: randomized controlled trial. JMIR Mhealth Uhealth 8, e19869. 10.2196/19869 33141092 PMC7671838

[B42] Zhihua S. Jialin S. Xiaoqu Z. (2017). Effects of low-carbohydrate diet nutrition intervention on glucose and lipid metabolism and pregnancy in patients with obese polycystic ovary syndrome. Chin. J. Phys. 19, 1209–1212. 10.3760/cma.j.issn.1008-1372.2017.08.022

